# Seasonal plasticity in photoprotection modulates UV‐induced *hsp* gene expression in copepods from a clear lake

**DOI:** 10.1002/lno.10793

**Published:** 2018-02-16

**Authors:** B. Tartarotti, A. Alfreider, M. Egg, N. Saul, T. Schneider, R. Sommaruga, A. Tischler, J. Vetter

**Affiliations:** ^1^ Lake and Glacier Research Group, Institute of Ecology University of Innsbruck Innsbruck Austria; ^2^ Ecophysiology, Institute of Zoology University of Innsbruck Innsbruck Austria; ^3^ Molecular Genetics Group, Institute of Biology Humboldt‐Universität zu Berlin Berlin Germany

## Abstract

Zooplankton from clear alpine lakes is exposed to stressful levels of solar UV radiation (UVR). As these pelagic organisms experience high UVR and large changes in solar radiation conditions between ice‐free and ice‐cover periods, they have evolved various strategies to minimize UVR exposure and damage. Here, we studied the relation between photoprotection levels (mycosporine‐like amino acids, carotenoids), antioxidant capacities, and gene expression of heat shock proteins (*hsps*) as indicator of stress in the copepod *Cyclops abyssorum tatricus* during the course of a year. Expression of *hsp60*, *hsp70*, and *hsp90* was measured in the field (baseline expression [BE]) and after UVR exposure in the laboratory. The BE differed among genes and seasons (*hsp60*: high during summer, *hsp70* and *hsp90*: high during the ice‐cover period). The gene expression of *hsp70* was upregulated after exposure to UVR (up to 5.2‐fold change), while *hsp60* and *hsp90* were only constitutively expressed. A strong seasonal pattern was found in the photoprotective compounds and antioxidant capacities, with highest levels during the ice‐free period. The extent of upregulation of *hsp70* gene expression increased with decreasing photoprotection levels and peaked 24 h post UVR exposure (9.6‐fold change) at the time of lowest photoprotection (February). Our data suggest that *hsp70* gene expression is modulated by seasonal plasticity in photoprotection. This ability of adequate stress response is essential for survival in highly variable ecosystems such as alpine lakes.

The exposure of zooplankton to solar UV radiation (UVR) depends largely on the transparency of the lakes which they inhabit (Rautio and Tartarotti 2010). Alpine lakes (i.e., lakes located above the treeline), with the exception of those fed by turbid meltwater, belong to the most UV‐transparent aquatic ecosystems worldwide (Morris et al. 1995; Sommaruga 2001). Copepods from these ecosystems are well adapted to their harsh environment, as they thrive in these cold, oligotrophic, and UV‐exposed lakes (Tartarotti et al. 1999, 2017; Kessler et al. 2008; Fischer et al. 2015). During the ice‐free season (∼ 4 months), UVR (290–400 nm) penetrates deep into the water column of these commonly shallow ecosystems (Laurion et al. 2000), while during ice‐cover periods this wavelength range is largely excluded (Belzile et al. 2002). *Cyclops abyssorum tatricus*, the dominant copepod species in lakes of the Eastern Alps, revealed high UVR tolerance when exposed to potentially lethal surface UVR levels during summer (Tartarotti et al. 1999). High tolerance to UVR was also shown in the otherwise highly UVR sensitive (Leech and Williamson 2000) cladoceran *Daphnia* when living in clear subarctic ponds (Zellmer et al. 2004). In clear lakes, copepods avoid UVR exposure by staying deep in the water column during the day (Tartarotti et al. 1999, 2017; Fischer et al. 2015) and they accumulate high levels of photoprotective compounds such as mycosporine‐like amino acids (MAAs) (Tartarotti et al. 2001, 2004, 2017; Persaud et al. 2007) or carotenoids (Hairston 1978; Tartarotti et al. 1999; Persaud et al. 2007; Sommaruga 2010). While UV‐absorbing MAAs are intracellular, water‐soluble, low molecular weight compounds absorbing in the UV wavelength region (309–360 nm) (Karentz et al. 1991), carotenoids such as astaxanthin have strong antioxidant properties (Krinsky 1979). UVR induces oxidative stress, thus various antioxidants are necessary for the neutralization and quenching of UV‐induced toxic photoproducts including reactive oxygen species (Vincent and Neale 2000). Photo‐protective compounds are known to vary seasonally in copepods (Moeller et al. 2005; Tartarotti and Sommaruga 2006; Persaud et al. 2007). In *C. abyssorum tatricus*, the concentrations of MAAs are three times higher during summer than during the period with ice‐cover (Tartarotti and Sommaruga 2006), indicating the importance of these compounds in protecting aquatic organisms against high solar radiation levels.

Avoidance, photoprotection, and DNA repair processes are only part of the suite of physiological, biochemical, and molecular defense responses that improve survival in high UVR environments such as clear alpine lakes. The rapid induction of highly conserved polypeptides known as stress proteins or heat shock proteins (Hsps) is a typical response of organisms to environmental challenges, including thermal, oxidative, chemical, and UV stress (Sanders 1993; Feder and Hofmann 1999; Sørensen et al. 2003; Richter et al. 2010). In general, Hsps are important in maintaining protein homeostasis (Hofmann et al. 2002), but they also function as molecular chaperones. As chaperones they stabilize and refold denatured proteins, conferring tolerance to harsh conditions such as high temperatures (Lindquist 1986) and prevent the formation of deleterious cytotoxic aggregates (Bucciantini et al. 2002). The expression of genes encoding Hsps is either constitutive, constitutive and inducible, or solely inducible (Sørensen et al. 2003). One of the first studies using genetic approaches to detect stress responses in copepods showed that both short‐ and long‐term thermal stress resulted in a fourfold induction of *hsp70* gene expression in *Calanus finmarchicus* (Voznesensky et al. 2004). Interspecific differences in gene expression (*hsp70* and *hsp90*) and the stress factors needed to induce a response (handling and heat) were observed in *Acartia tonsa* and *Eurytemora affinis* (Rahlff et al. 2017). Recently, upregulation of *hsp* gene expression was reported in *Tigriopus japonicus* and *Paracyclopina nana* after UV‐B exposure (Kim et al. 2015; Won et al. 2015). Kim et al. (2015) suggested that small *hsps*, *hsp70*, and *hsp90* may be part of the cellular protection against UV‐B radiation. In a recent study, Han et al. (2016) found species‐specific and gene‐specific variability in the mRNA expression pattern of different *hsp* genes after UVR exposure. In two congeneric *Tigriopus* species, the Antarctic *T. kingsejongensis* showed upregulation of the expression of small, but not large *hsp* genes, while in the temperate *T. japonicus* the gene expression of all *hsp*s increased in response to UVR, resulting in better survival and greater adaptiveness (i.e., upregulation of antioxidant defense genes) in the latter species.

Depending on the content of photoprotective compounds such as MAAs in copepods, there might be a trait compensation in *hsp* expression and photoprotection and antioxidant capacities over the seasons. In the present study, our aim was to investigate how copepods with varying levels of photoprotection respond to the effects of UVR at the gene expression level. We hypothesized that stress proteins will be upregulated to account for the need for readily available protection to maintain cellular homeostasis and that the extent of upregulation will increase with decreasing photoprotection levels present in the copepods. To test these hypotheses, we measured the concentrations of photoprotective compounds (MAAs, carotenoids) and antioxidant capacities and did laboratory experiments to assess UV‐induced *hsp60*, *hsp70*, and *hsp90* gene expression in *C. abyssorum tatricus* during the course of a year. Our results indicate that adaptive variation in photoprotection levels modifies the molecular response to environmental stressors such as UVR.

## Materials

### Sampling

We collected the cyclopoid copepod *C. abyssorum tatricus* Kozminski (Einsle 1993) from the alpine lake Gossenköllesee (elevation 2413 m a.s.l., *Z*_max_ 9.9 m, lake area 1.7 ha, 10% attenuation depth at 320 nm ∼ 9.6 m) between August 2013 and July 2014 on a two‐monthly basis. The lake was covered with ice from November until mid‐June. Copepods were obtained by making several single net (mesh size: 50 μm) vertical tows at the center of the lake at dusk. Immediately after sampling, copepods (60 copepods per sample; triplicates) were flash frozen in liquid nitrogen to obtain the stress protein gene expression of the copepods present in the natural environment (baseline expression [BE]). For the samples, copepodid CII‐CIV life stages were used; however, the majority of the individuals were copepodid CIV as this developmental stage was present in relatively high abundances year‐round. Previous studies have also shown that copepodid CIV is the dominant developmental stage in this lake during most of the year (Eppacher 1968; Praptokardiyo 1979). No nauplii or adult copepods were used. The time from collection to preservation of the samples was less than 30 min (sorting ≤ 10 min per sample). In addition, copepod samples (triplicates; see above) were flash frozen for antioxidant capacity measurements. Live copepods (same sampling procedure as described above) were maintained at in situ temperature conditions and fed with *Cryptomonas* upon return to the laboratory in Innsbruck. After ∼ 48 h of acclimation, the UV exposure experiments were done.

### UV exposure experiments

Before the experiments started, copepods (triplicate samples; see above) were flash frozen to determine background levels of the *hsp* gene expression in *C. abyssorum tatricus* (hereafter time zero, *t*_0_). For the experiments, copepods were carefully transferred into quartz glass tubes (250 mL of 10 μm mesh‐filtered lake water and ∼ 60 copepods per tube). The copepods (triplicates per treatment) were exposed to UVR plus photo‐reactivating radiation (UV) or to photo‐reactive radiation (PAR) alone (tubes covered with vinyl chloride foil, 50% transmittance at 405 nm) using four A‐340 Q‐Panel lamps (Q‐Panel) and two white daylight lamps (F36W/860; General Electric Lightning). The distance between the lamps and tubes was 25 cm, resulting in irradiance values of 1.4 W m^−2^ (integrated between 280 nm and 320 nm; *see* Sommaruga et al. 1996 for further details). Quartz tubes covered with aluminum foil served as dark control (D; triplicates). The experiments were run in an environmental chamber set at ambient temperature (4–8°C; depending on the seasonal variation in the average water temperature on the day of sampling) for an exposure period of 6 h. This period was chosen to simulate realistic exposure conditions in terms of duration and UVR dose. Although the copepods are protected from UVR in their natural environment during the ice‐cover season, we wanted to follow the UVR response of these organisms also at times of low photoprotection. At the end of the exposure period, copepods were placed in the dark for 1 h (recovery period after relief of stress; Rhee et al. 2009) before they were flash frozen. Another set of animals (triplicates per treatment and control) was kept in dark conditions for 24 h to follow the post‐UV exposure stress response (post‐UV exposure). Copepods from all treatments were checked for mortality under a stereo microscope (Olympus SZ40), flash frozen in liquid nitrogen (starting with the UV treatment; sorting ≤ 10 min per sample) and stored at −80°C until the extraction of RNA.

### Extraction of RNA and cDNA synthesis

TRIzol reagent (400 μL; Invitrogen) was used to extract total copepod RNA following the manufacturer's instructions. An Ultra Turrax (T10, IKA) disperser was used to homogenize the samples. gDNA eliminator solution (44.4 μL; Qiagen) was added to the homogenate to eliminate potential contamination with genomic DNA. The crude RNA extract (220 μL) was transferred to a 2 mL microcentrifuge tube, and 220 μL of 70% ethanol (Electran) were added and mixed thoroughly by pipetting. The copepod RNA was further purified (RNeasy Mini Kit, Qiagen; according the manufacturer's protocol) and a volume of 30 μL water (RNase‐free) was used to elute the RNA.

Two microliters of total RNA were measured with a NanoDrop (ND1000, Thermo Fisher Scientific) to check for RNA quality (mean A260/280 ratio: 2.13 ± 0.05) and 1 μL was run on a 1.2% agarose gel (Certified Molecular Biology Agarose, Biorad) stained with GelRed (Biotium) at 90 V for 1 h to check the RNA integrity. The RNA concentration was quantified in triplicate with a plate reader (2030 Multilabel Plate Reader Victor X4, Perkin Elmer) and the Quant‐iT RiboGreen Assay Kit (Life Technologies). For first‐strand complementary DNA (cDNA) synthesis, 450 ng total copepod RNA and random hexamer primers (#S0142, Thermo Scientific) were used. cDNA was synthesized using MMLV H minus reverse transcriptase (#EP0452, Thermo Scientific) following the manufacturer's protocol with slight modifications by using less reverse transcriptase. No reverse transcriptase controls were included to test for potential genomic DNA contamination. The first‐strand cDNA (total volume 50 μL) was frozen at −20°C before quantitative polymerase chain reaction (qPCR) analysis.

### Primer design and cloning

In order to amplify partial *hsp* sequences of *C. abyssorum tatricus*, degenerate primers were designed targeting conserved regions identified by sequence alignment of *hsp60*, *hsp70*, and *hsp90* genes from several crustacean and other arthropod species. Sequences were aligned using MUSCLE algorithm (MEGA 6.0 software; Tamura et al. 2013) and visual inspection of the alignment. PCR was done using GoTaq^®^ G2 Hot Start Master Mix (Promega, Madison, Wisconsin, U.S.A.) using standard procedure recommended by the manufacturer. The following PCR conditions were used: 95°C/4 min; 40 cycles of 94°C/15 s, 50–55°C gradient annealing temperatures/30 s, 68°C/7 min; hold at 4°C. Products were visualized on 1.5% agarose gels. Distinct bands of correct size were cut out of the agarose gel and DNA fragments were purified using a gel extraction kit (MinElute^®^, Qiagen, Valencia, California). For cloning purpose, PCR products were ligated into vector plasmid pGEM‐T‐Easy and JM109 *Escherichia coli* cells were used for transformation following the instructions by the manufacturer (Promega, Madison, Wisconsin, U.S.A.). Clones were investigated for the occurrence of proper *hsp* DNA fragments by PCR using vector‐specific M13 primers flanking the insert. Selected amplificates of *hsp60*, *hsp70*, and *hsp90* partial genes were sent for sequencing by means of a Sanger sequencing platform (Eurofins MWG Operon, Ebersberg, Germany). *Hsp* nucleotide and deduced amino acid sequences similarity searches were performed using NCBI's tools BLASTN and BLASTP (Altschul et al. 1990). Sequences used for subsequent qPCR primer design have been deposited in GenBank database under accession numbers KY792996 (*hsp60*), KY792997 (*hsp70)*, and KY792998 (*hsp90*).

### qPCR

Oligonucleotide primers were designed to target ∼ 60 bp amplicons using Primer Express Software 3.0 (Thermo Fisher Scientific, Waltham, Massachusetts, U.S.A.). Primer sequences and PCR amplification efficiencies for the respective genes are as follows: *hsp60* forward [f]: GGCTGGAGACGGTACCACAA, reverse [r]: ACCTGCCTTGGCAATTGC; efficiency [e]: 92%; *hsp70* f: CAACCAGAAGCAGGGAAAGAAG, r: CCACCCCCGAGGTCAAA; e: 96%, and *hsp90* f: AACATCAAGCTTGGTATCCATGAA, r: GAGGAGCCCGGCTAACTTCT; e: 96%. All qPCR reactions were run in a QuantStudio3 real‐time PCR detection system (Thermo Fisher Scientific). PCR was run in triplicate technical repeats, using 10 *μ*L volumes of a mixture comprising 1× PowerSybr Green PCR Master Mix (Thermo Fisher Scientific), forward and reverse qPCR‐specific primer (500 nM each), nonacetylated bovine serum albumine (0.25 *μ*g; Sigma‐Aldrich), and 1 *μ*L cDNA. We included controls of RNA incubated with random hexamer primers but without reverse transcriptase (no RT). In addition, to test for amplification variability between runs, we also included an internal control (pooled sample of UV‐stressed and unstressed *C. abyssorum tatricus* cDNA). The qPCR conditions were as follows: 50°C/2 min; 40 cycles of 95°C/15 s, 60°C/1 min. Data acquisition and analysis were performed with the QuantStudio^™^ Design and Analysis Software v1.4.1 (Thermo Fisher Scientific). Serial dilutions of gene‐specific quantified *C. abyssorum tatricus* cDNA were made for the determination of real‐time PCR efficiency. Absolute copy numbers (absolute quantification method) were calculated by plotting the CT values vs. the log10 of the initial copy numbers, quantified with the Quant‐iT Picogreen dsDNA Assay Kit (Life Technologies), and the specific molecular weight of each amplicon. The copy numbers were normalized to 10 ng of total copepod RNA. To ensure that only a single product was amplified and no primer dimers were formed, PCR products were subjected to melt‐curve analysis (conditions: 95°C/15 s, 60°C/1 min, 95°C/1 s) after amplification. In addition, selected products were run on 1.2% agarose gels, consistently yielding one single band.

### MAAs, carotenoids, and antioxidant capacities

Copepods for analyses of MAAs and carotenoids were kept in the dark at 4–8°C and processed within 24 h. Fifteen (MAAs) and 30 (carotenoids) narcotized (CO_2_) copepods (mainly copepodid CII to CIV life stages, no nauplii and egg‐carrying females; care was taken to use a similar life stage distribution among sampling periods) were separated into microcentrifuge tubes. The samples (triplicates) were immediately frozen at −80°C. We extracted MAAs according to Tartarotti et al. (2017). Briefly, extraction was done in 400 μL of aqueous methanol (25% v/v; MeOH) in a water bath for 2 h at 45°C. Samples were sonicated on ice at the beginning of the extraction (30 s at 40 W; UP 200S, Dr Hielscher GmbH, Germany) and stored overnight at −80°C for characterization using isocratic reverse‐phase high performance liquid chromatography (HPLC). To separate and quantify MAAs, aliquots (80 μL) were injected into a Phenosphere 5 μm RP‐8 column (4.6 mm internal diameter × 25 cm, Phenomenex) protected with a RP‐8 guard column (Brownlee). The mobile phase of 0.1% acetic acid in 25% aqueous MeOH (v/v) had a flow rate of 0.75 mL min^−1^. We used online UV spectroscopy in a Dionex poly‐diode array detector to detect the MAAs in the eluate. Identification of individual peaks was based on relative retention time, absorption spectra, and co‐chromatography with known standards (*Porphyra tenera*). We then calculated the content of specific MAAs in each sample from peak areas at 310 nm, 320 nm, 334 nm, and 360 nm, using published molar extinction coefficients (*see* Tartarotti et al. 2001). MAA concentrations were normalized to the dry weight of the copepods and were expressed as [μg mg^−1^ dry weight]. For dry weight estimation, the *C. abyssorum tatricus* body length was measured at a magnification of ×100 and biomass was then calculated according to Praptokardiyo (1979). Carotenoids were extracted in 400 μL 95% ethanol at 8°C for 14 h. The samples were sonicated on ice for 1 min (40 W) at the beginning of the extraction. Carotenoids were analyzed using a double‐beam spectrophotometer (Hitachi U‐2000) against an ethanol blank. The concentration of carotenoids was calculated as in Hairston (1978) and expressed as [μg mg^−1^ dry weight].

Antioxidant capacities were measured as described in Tartarotti et al. (2014). Briefly, the copepods were cleaved in a Speedmill (Analytik Jena, Germany) followed by centrifugation (11,600 × *g*, 3 min) using a sodium hydrogen phosphate buffer (0.1 M, pH 6.5). The cooled supernatant was directly used to determine the antioxidant capacity of water‐soluble antioxidants (e.g., ascorbic acid, glutathione, uric acid, and lipoic acid) or was further processed for the extraction of lipid‐soluble antioxidants (e.g., β‐carotene, tocopherols, vitamins A, D, steroids, and aromatic substances) following Bligh and Dyer (1959). Antioxidant capacities were analyzed based on Popov and Lewin (1999) in a PhotoChem device (Analytik Jena, Jena, Germany) via photo‐chemiluminescence. Copepod protein content was measured according to Bradford (1976), and antioxidant capacities were expressed as nM trolox or ascorbic acid equivalents [mg protein]^−1^ for lipophilic and hydrophilic antioxidants, respectively.

### Data treatment

We report all data as mean ± SD; level of significance was set to *p* < 0.05. The significance of differences in *hsp* gene expression was evaluated by one‐way analysis of variance (ANOVA) followed by Tukey honestly significant difference (HSD) post hoc test. For the *hsp* genes, copy numbers were log (natural logarithm, ln) transformed. Differences in photoprotection levels and *hsp* BE between ice‐free and ice‐cover periods were analyzed with *t*‐tests. We ran all statistical analyses using the software package Statistica (Version 12).

## Results

### *Hsp60, Hsp70*, and *Hsp90* gene expression

**Figure 1:**
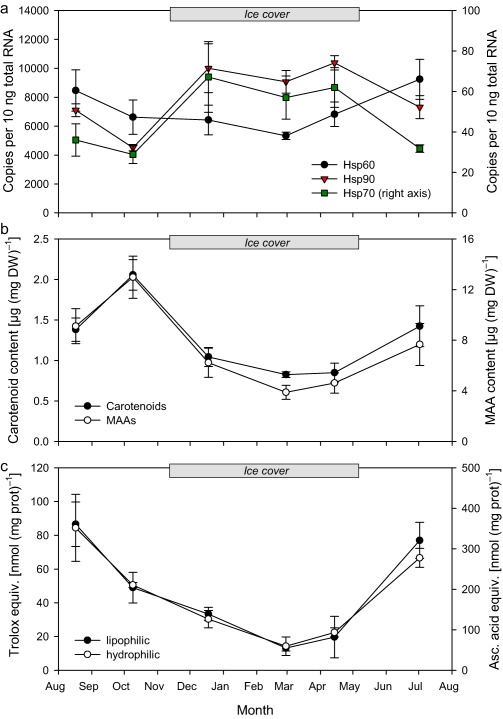
Seasonal variation of (**a**) *hsp60* (black circles), *hsp70* (green squares), and *hsp90* (red triangles) BE (copies per 10 ng total RNA; *n* = 3), (**b**) total mean MAA (white circles) and carotenoid (black circles) concentrations (μg mg^−1^ dry weight; *n* = 3), and (**c**) mean lipophilic (nmol trolox equivalents mg protein^−1^; *n* = 3; black circles) and hydrophilic antioxidant capacity (nmol ascorbic acid equivalents mg protein^−1^; *n* = 3; white circles) in *C. abyssorum tatricus* from August 2013 to July 2014. Error bars indicate ±1 SD.

**Figure 2:**
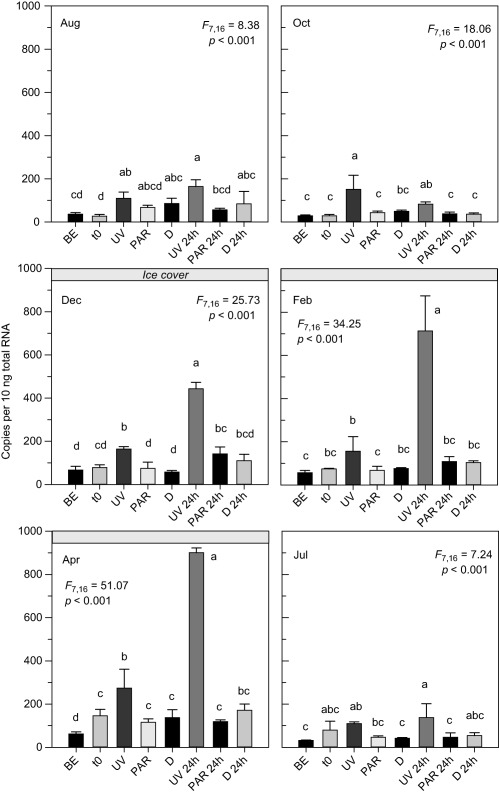
Expression of heat shock protein 70 (*hsp70*) gene in *C. abyssorum tatricus* over the study period from August 2013 to July 2014. Gene expression was quantified (absolute quantification method) in the natural population (BE), at the beginning of the experiment (*t*_0_), following 6 h of UVR exposure with photo‐reactivation radiation (UV), 6 h of photo‐reactivation radiation (PAR), when kept in the dark (D), and 24 h post UVR exposure (UV 24 h, PAR 24 h, and D 24 h); *n* = 3 biological replicates, 60 copepodid CII to CIV life stages were pooled per sample. Shown are mean +1 SD expression. Different letters above the bars indicate a significant difference found with one‐way ANOVA with Tukey HSD post hoc test.

**Figure 3:**
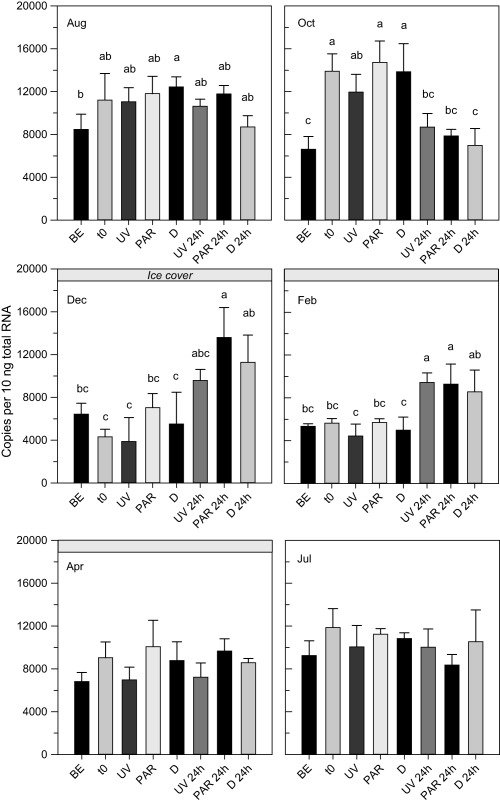
Expression of heat shock protein 60 (*hsp60*) gene in *C. abyssorum tatricus* over the study period from August 2013 to July 2014. Gene expression was quantified (absolute quantification method) in the natural population (BE), at the beginning of the experiment (*t*_0_), following 6 h of UVR exposure with photo‐reactivation radiation (UV), 6 h of photo‐reactivation radiation (PAR), when kept in the dark (D), and 24 h post UVR exposure (UV 24 h, PAR 24 h, and D 24 h); *n* = 3 biological replicates, 60 copepodid CII to CIV life stages were pooled per sample. Shown are mean + 1 SD expression. Different letters above the bars indicate a significant difference found with one‐way ANOVA with Tukey HSD post hoc test.

**Figure 4:**
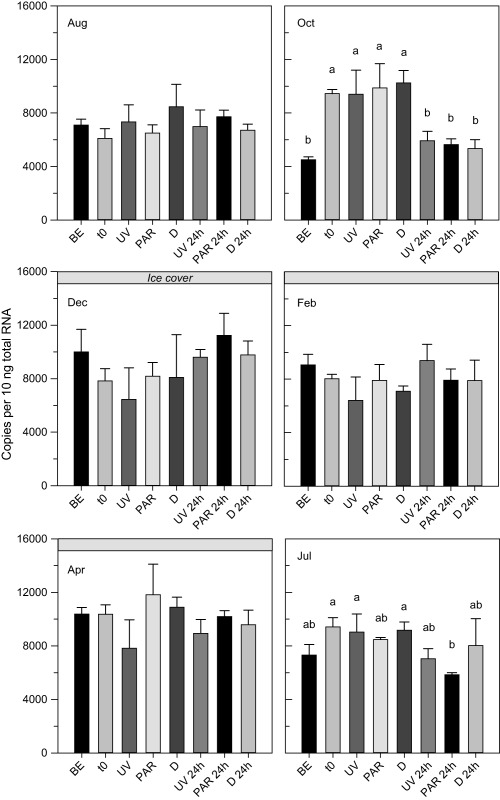
Expression of heat shock protein 90 (*hsp90*) gene in *C. abyssorum tatricus* over the study period from August 2013 to July 2014. Gene expression was quantified (absolute quantification method) in the natural population (BE), at the beginning of the experiment (*t*_0_), following 6 h of UVR exposure with photo‐reactivation radiation (UV), 6 h of photo‐reactivation radiation (PAR), when kept in the dark (D), and 24 h post UVR exposure (UV 24 h, PAR 24 h, and D 24 h); *n* = 3 biological replicates, 60 copepodid CII to CIV life stages were pooled per sample. Shown are mean + 1 SD expression. Different letters above the bars indicate a significant difference found with one‐way ANOVA with Tukey HSD post hoc test.

BLAST searches of the *C. abyssorum tatricus* deduced *hsp60*, *hsp70*, and *hsp90* amino acid sequences resulted in high similarity (80–90%) to corresponding sequences from other copepods such as *P. nana*, *T. japonicus*, and *Eurytemora pacifica*. The constitutive expression of the *hsp* genes followed different seasonal patterns (Fig. 1a). *Hsp60* levels were significantly higher during the ice‐free period than at times when the lake was ice‐covered (*t*‐test; df = 16, *t* = 3.01, *p* = 0.008). In contrast, *hsp70* and *hsp90* levels were significantly lower during the ice‐free period (*t*‐tests; *hsp70*: df = 16, *t* = −7.77, *p* < 0.001; *hsp90*: df = 16, *t* = −5.12, *p* < 0.001). The *hsp* gene expression was similar to the baseline levels at the beginning of the experiments (*t*_0_) (Figs. 2, 3, 4), indicating low handling stress effects. The only exceptions were the October (*hsp60* and *hsp90*) and April (*hsp70*) experiments, where levels of gene copy numbers were higher at *t*_0_ (Figs. 2, 3, 4). No UVR‐induced mortality (< 5%) was observed in the copepods. Although the three *hsp* genes were expressed in all samples, only the mRNA expression pattern of the *hsp70* gene increased significantly in response to UVR (Fig. 2). The *hsp60* gene expression showed some variation in four out of the six experimental runs; however, there was no consistent pattern of up‐ or downregulation in a specific treatment (Fig. 3). Similarly, the *hsp90* gene expression showed some, but even less variation than *hsp60* (Fig. 4). In no case, an induction of these genes was detected after exposure to UVR. During the whole study period, the *hsp70* gene expression in *C. abyssorum tatricus* was highest in the UV and 24 h post‐UV exposure treatments (Fig. 2). The *hsp70* gene expression after 6 h of UVR exposure was similar during the ice‐free season (August, October, and July in the following year) and the first two dates with ice‐cover (December and February), while in April a significantly higher *hsp70* expression was observed. The maximum *hsp70* gene expression was found 24 h post‐UV exposure, except for the October experiment where gene expression was higher in the 6 h UVR treatment (Fig. 2).

### MAAs, carotenoids, and antioxidant capacities

The concentration of MAAs in the copepods changed over the season with high levels during the ice‐free period and a decrease when the lake was ice‐covered (up to 3.4‐fold difference; *t*‐test; df = 16, *t* = 4.97, *p* < 0.001) (Fig. 1b). Lowest MAA levels were present at the time of the thickest ice‐cover (February and April). Similarly, the carotenoid concentrations varied over the study period with up to 2.5 times higher concentrations during the ice‐free than during the ice‐covered period (*t*‐test; df = 16, *t* = 5.44, *p* < 0.001; Fig. 1b). The lowest carotenoid contents were again observed in February and April. MAAs and carotenoids were highly positively correlated (*r* = 0.983, *n* = 6, *p* < 0.001). A seasonal trend was also seen in the lipophilic and hydrophilic antioxidant capacities (Fig. 1c). Highest levels were found during the ice‐free period in July 2013 and August 2014, while lowest levels were again observed in February with up to 6.6‐ and 5.9‐fold difference for lipophilic (*t*‐test; df = 15, *t* = 6.23, *p* < 0.001) and hydrophilic (*t*‐test; df = 16, *t* = 6.84, *p* < 0.001) antioxidant capacities, respectively.

### Relationship between photoprotection and gene expression

At times of low photoprotection (i.e., when the lake was ice‐covered), *C. abyssorum tatricus hsp70* gene expression was highest in both the UVR exposure and the 24 h post‐UVR exposure treatments (Fig. 2). However, relative to the expression at the beginning of the experiment (*t*_0_), the extent of *hsp70* upregulation after UVR exposure was highest in August and October (greater than fourfold) and approximately twofold on the other dates. In the 24 h post‐UV exposure treatment, the strongest upregulation of gene expression (9.6‐fold) was found in February, when antioxidants and photoprotective compounds in *C. abyssorum tatricus* were lowest. Conversely, at the time of highest MAA and carotenoid levels (October), only a slight upregulation of the *C. abyssorum tatricus hsp70* gene expression 24 h post‐UV exposure was observed (Fig. 2).

## Discussion

Zooplankton living in clear alpine lakes are exposed to high levels of solar UVR, which is an important environmental stress factor in these ecosystems (Sommaruga 2001; Rose et al. 2009). While seasonal variations in photoprotective MAA concentrations have already been observed in an earlier study (Tartarotti and Sommaruga 2006), this is the first report of seasonal changes in carotenoid concentrations and antioxidant capacities in alpine copepods. The annual pattern with maximum concentrations at times of high incident solar UVR levels is consistent with previous observations, suggesting that MAAs are accumulated in response to UVR‐induced stress (Moeller et al. 2005; Tartarotti and Sommaruga 2006; Persaud et al. 2007). However, high summer carotenoid levels seem to be an adaptive response of cyclopoid copepods from clear alpine lakes, whereas studies in lower‐elevation lakes have shown relatively low concentrations during summer as compared to winter‐spring (Hansson 2004; Moeller et al. 2005; Persaud et al. 2007; Schneider et al. 2016). Schneider et al. (2016) found that carotenoid accumulation in a calanoid copepod, *Leptodiaptomus minutus*, from a low‐UVR boreal brown‐water lake is related to the copepod life cycle and lipid metabolism. In clear lakes, the accumulation of carotenoids protects copepods from potential photooxidative damage. Thus, the role of these pigments seems to depend on the UVR transparency of the study system. Here, we link the seasonal variation in photoprotection levels to the UVR susceptibility of *C. abyssorum tatricus* at the molecular level. During the ice‐free period when photoprotection levels are high in these organisms, *hsp70* gene expression was upregulated after UVR exposure, but the response is relatively weak (Fig. 2). However, during the ice‐cover season, when UVR generally is not transmitted through ice (Belzile et al. 2002), *hsp70* gene expression is high and peaks at the time of lowest photoprotection (Fig. 1b,c). Due to the plasticity in photoprotection, we assume that the copepods have different UVR sensitivity, leading to the same treatment (i.e., 6 h UV exposure) being differently stressful and thus resulting in different *hsp* response of the organism. *Hsp70* are the most commonly upregulated of all *hsp*s (Lewis et al. 1999) and protect copepods in response to multiple stressors including UVR (Won et al. 2015). The upregulation of mRNA expression, an early sign of stress, and its relation to the photoprotection level of the copepods further supports the idea that high levels of MAAs, carotenoids, and antioxidant capacities are important in protecting *C. abyssorum tatricus* from UVR damage.

The response to stress at the transcriptional level typically occurs in copepods within 20 min (heat stress; Rhee et al. 2009) to 1 h (UVR stress; Han et al. 2016). Depending on the UVR dose, the change in *hsp70* gene expression in the marine copepod *T. japonicus* ranges between onefold (i.e., no change) and eightfold change (Kim et al. 2015). The changes in gene expression we found in *C. abyssorum tatricus* after 6 h of UVR exposure were in a similar range (1.4–5.2‐fold expression changes compared to *t*_0_, Fig. 2). The persistence of response may be important to meet increased chaperoning needs at times of stress. For example, extended (18 h) presence of *hsp70* mRNA was observed in the mussel *Mytilus californianus*, experiencing daily extreme thermal stress during low tide (Hofmann et al. 2005). In the copepod *P. nana*, expression of *hsp* genes is highest 6 h post UVR exposure, but the expression of small hsps (*hsp10*) is still high 12 and 24 h post UVR (Won et al. 2015). Surprisingly, in *C. abyssorum tatricus* highest *hsp70* gene expression levels were observed 24 h post UVR exposure (Fig. 2), with strong seasonal variation in expression levels (1.6–9.6‐fold change), confirming the importance of *hsp70* in UV protection.

The induction of *hsp* gene expression is not genetically hardwired, but depends on the recent exposure history of an organism and on acclimatization and acclimation (Dietz and Somero 1992; Dietz 1994). In coastal fish, *Gillichthys* spp., higher HSP90 levels (protein level) were found in brain tissue of summer‐ compared to winter‐acclimatized individuals (Dietz and Somero 1992). Seasonal variations of HSPs have been also reported in invertebrates such as marine mussels, *Mytilus* spp. (Hofmann and Somero 1995; Chapple et al. 1998; Minier et al. 2000), and the sea anemone *Anemonia viridis* (Choresh et al. 2001), with high levels during summer. Lejeusne et al. (2006) observed that the BE of HSPs in the marine cave mysid, *Hemimysis margalefi*, was affected by natural temperature fluctuations. In summer, HSP60 (protein level) and to a lesser extent HSP50 expression was higher than in winter, while no correlation between HSP90 and seasonal variations of temperature was found (Lejeusne et al. 2006). In all of these studies, fluctuations in HSP expression were strongly related to temperature variations. Such seasonal changes in *hsp* expression underline the ecological importance of these genes in natural populations (Sørensen et al. 2003). The elevated HSP levels during summer may indicate higher risk of protein damage at high temperature (Dietz and Somero 1992; Lejeusne et al. 2006). In *C. abyssorum tatricus*, the *hsp60* BE level was higher during the ice‐free than the ice‐covered period (*p* = 0.08). During summer, this constitutively expressed gene had the highest copy numbers of the three genes measured (Fig. 1a). Although water temperatures peak during the summer months (up to ∼ 15°C at the surface), the copepods stay close to the bottom of the lake at 4–6°C during daytime (Tartarotti et al. 1999, 2017). However, they migrate to upper, warmer water layers during night (Eppacher 1968), experiencing temperature differences of up to 11°C. During the long ice‐cover period (∼ 8 months), they are exposed to relatively stable, low temperatures (0–4°C). BE levels of *hsp70* and *hsp90* did not follow the same trend and were higher during the ice‐cover season (*p* < 0.001) (Fig. 1a). These differential expressions may be caused by gene‐specific responses, as shown in *H. margalefi* (Lejeusne et al. 2006), and may depend on the cellular function of the different *hsp*s genes. The higher levels of *hsp60*, a mitochondrial gene, during the ice‐free season might reflect the metabolically more active period of the year. However, high levels of a specific *hsp* may also be an adaptive strategy to the environment without relation to recent stress exposure or the stress status of the organism (Morris et al. 2013). Although rarely studied, cold‐induced denaturation of proteins (< 10°C) can be found in eukaryotic cells, which may pose a challenge to protein homeostasis similar to thermal stress (Kandror et al. 2004). In addition to organisms from “extreme” but stable environments, high constitutive HSP70 expression has also been observed in organisms adapted to highly variable conditions (Morris et al. 2013). While alpine lakes are stable during the ice‐cover season, they are a highly variable environment (e.g., temperature gradients) for migrating organisms such as copepods during the ice‐free period. Very recently, rhythmic expression of circadian clock genes was found in the marine copepod *C. finmarchicus* during diel vertical migration (Häfker et al. 2017). Although so far, diel variation in *hsps* has not been reported in copepods, one can speculate that diurnal variability may be also present in these genes, as shown for HSP70 in the marine mollusk *Acanthopleura granulate* (Schill et al. 2002).

In any particular species, not necessarily all *hsp* family genes respond to stress. For example, in the marine copepods *T. japonicus* and *A. tonsa*, *hsp70* but not *hsp90* expression is induced by heat stress (Rhee et al. 2009; Rahlff et al. 2017). Similarly, only constitutive but not induced levels were observed for *hsp60* and *hsp90* in *C. abyssorum tatricus* after UV stress (Figs. 3, 4). The expression of *hsp70* may provide sufficient protection against UV‐induced protein damage, alleviating the need for expression of other molecular chaperones. Moreover, the induction of *hsps* may be costly (Sørensen et al. 2003), which led Rhee et al. (2009) to speculate that *T. japonicus*, a copepod well‐adapted to conditions of high temperature, salinity, and UV stress (i.e., rock pools of the intertidal zone), reduces metabolic costs by inducing only one type of *hsp*. However, in copepods, such a cost‐saving strategy remains to be confirmed by further studies because upregulation of a single gene might also be costly if more of that gene product is required to compensate for other *hsp*s. Alternatively, *C. abyssorum tatricus* from alpine lakes exposed to low temperatures most of the year may have lost the *hsp60* and *hsp90* stress response. For example, loss of the heat‐shock response (HSP70) was described in the Antarctic fish, *Trematomus bernacchii*, living at stable, low temperatures (Hofmann et al. 2000). Another explanation is that adaptation through other means (e.g., photoprotection) might reduce the need for molecular stress responses. Interestingly, in many studies on copepods upregulation of *hsp70* gene expression after stress exposure was shown, while the expression of other *hsp* genes seems more variable (Rhee et al. 2009; Petkeviciute et al. 2015; Won et al. 2015; Rahlff et al. 2017; this study).

Several members (i.e., isoforms) of the *hsp70* family are potentially present in a given species (Morris et al. 2013). Ideally, all members should be characterized and analyzed to determine which genes regulate the study organisms' stress level. However, when not working on model organisms, the analysis of all *hsp70* isoforms is highly time‐consuming (Morris et al. 2013). As 39 and 88 *hsp70* genes were discovered in sea urchins and the Pacific oyster, *Crassostrea gigas* (Zhang et al. 2012), there probably are various *hsp* isoforms also present in copepods. While the *hsp70* gene in our study is found in its constitutive and inducible form (Fig. 2), *hsp60* and *hsp90* were only constitutively expressed in the copepods (Figs. 3, 4). The high similarity to sequences of marine copepods, where *hsp* expression is induced after stress (Won et al. 2015), suggests that we did not isolate purely constitutive representatives of *hsp60* and *hsp90* families.

Life stage and gender may also influence the expression of *hsp*s. Gender‐specific expression of stress tolerance was found in adults of the copepod *E. affinis*, with maximal *hsp* transcript levels in ovigerous females (Boulangé‐Lecomte et al. 2014). To avoid the potentially confounding factor of life stage and gender‐specific differences in stress evaluation, we did not use larval stages (i.e., nauplii) or adult *C. abyssorum tatricus* in our experiments. We had to pool life stages as high numbers (1260 per experiment) of these tiny copepods (∼ 1 mm) were required. Although there is no study on the *hsp* gene expression in different copepodid life stages available, we assume that by using similar life stages (only copepodids) and life stage distribution (mainly CIV copepodids) the gene expression is not influenced by life stage. Another factor that should be taken into account when using natural populations for gene expression experiments is potential handling stress. In the copepod *C. finmarchicus*, expression of three transcripts (*hsp21*, *hsp22*, *hsp70A*) was induced by handling (Aruda et al. 2011). After sampling, we let the copepods acclimate for ∼ 48 h before the beginning of the experiments (*t*_0_). This time was sufficient to exclude potential handling stress as the gene expression of the stress‐inducible *hsp70* at *t*_0_ was, with one exception, similar to the baseline level (Fig. 2).

## Conclusion

In conclusion, the transcriptional regulation of *hsp* gene expression allows insight into how freshwater zooplankton and in particular *C. abyssorum tatricus* respond to UVR stress at the molecular level. Our study revealed significant changes in gene expression profiles (upregulation of *hsp70*, no change of *hsp60* and *hsp90* gene expression) following UVR exposure and showed that the extent of *hsp* gene expression is related to the photoprotection levels in the copepods, with stronger upregulation at times of low MAA and carotenoid concentrations and antioxidant capacities. Thus, copepods from clear alpine lakes not only efficiently utilize photoprotection and DNA repair mechanisms (Tartarotti et al. 2014), but also induce the *hsp* stress response when cellular damage accumulation is high. Future research should be directed toward field studies to see how these and other planktonic organisms respond in their natural environment and to reveal the diversity of strategies used to thrive in these highly variable habitats.

## Conflict of Interest

None declared.
